# Hsp90 can Accommodate the Simultaneous Binding of the FKBP52 and HOP Proteins

**DOI:** 10.18632/oncotarget.225

**Published:** 2011-02-28

**Authors:** Zacariah L. Hildenbrand, Sudheer K. Molugu, Nadia Herrera, Citlally Ramirez, Chuan Xiao, Ricardo A. Bernal

**Affiliations:** ^1^ Department of Chemistry, University of Texas at El Paso, 500 W. University Ave, El Paso, Texas 79968, U.S.A.; ^2^ Border Biomedical Research Center, University of Texas at El Paso, 500 W. University Ave, El Paso, Texas 79968, U.S.A.

**Keywords:** Hsp90, HOP, FKBP52, p23, Androgen Receptor Activation, hormone-dependent cancer, molecular chaperone

## Abstract

The regulation of steroidogenic hormone receptor-mediated activity plays an important role in the development of hormone-dependent cancers. For example, during prostate carcinogenesis, the regulatory function played by the androgen receptor is often converted from a growth suppressor to an oncogene thus promoting prostate cancer cell survival and eventual metastasis. Within the cytoplasm, steroid hormone receptor activity is regulated by the Hsp90 chaperone in conjunction with a series of co-chaperone proteins. Collectively, Hsp90 and its binding associates form a large heteromeric complex that scaffold the fully mature receptor for binding with the respective hormone. To date our understanding of the interactions between Hsp90 with the various TPR domain-containing co-chaperone proteins is limited due to a lack of available structural information. Here we present the stable formation of Hsp90_2_-FKBP52_1_- HOP_2_ and Hsp90_2_-FKBP52_1_-p23_2_-HOP_2_ complexes as detected by immunoprecipitation, time course dynamic light scattering and electron microscopy. The simultaneous binding of FKBP52 and HOP to the Hsp90 dimer provide direct evidence of a novel chaperone sub-complex that likely plays a transient role in the regulation of the fully mature steroid hormone receptor.

## INTRODUCTION

The activation of steroid hormone receptors plays an important role in the development of hormone-dependent cancers. For example, in a healthy prostate the androgen-bound receptor functions through a reciprocal paracrine interaction between the epithelial and stromal cells which activates a transcriptional cascade that ultimately stimulates the proliferation of epithelial cells [1]. Under physiological conditions, ligand-bound androgen receptor prevents the overgrowth of the epithelial compartment by suppressing cell proliferation and by triggering cellular differentiation [1-4]. However, during prostate carcinogenesis, the regulatory function played by the androgen receptor is often converted from a growth suppressor to an oncogene which in turn stimulates prostate cancer cell survival and proliferation [1, 5, 6].

Within the cytoplasmic milieu, steroid hormone receptor activity is tightly regulated by a series of chaperones that stabilize the receptor and maintain its ability to bind hormone [7-10]. These primarily include the Heat shock family of proteins Hsp40, 70 and 90, of which Hsp90 plays the most pivotal role in the later stages of hormone receptor maturation [11, 12]. Hsp90 is the most abundant molecular chaperone in the cytosol comprising roughly 1-2% of the cytosolic protein fraction [13]. Hsp90 binds with its client and associate proteins through short hydrophobic peptide sequences and facilitates protein folding through the shuffling between multiple ATP-dependent conformations [14, 15]. This activity is dependent on the dimeric nature of Hsp90, which is mediated by a high-affinity dimerization domain in the carboxy-terminal end of the protein [16, 17].

Hsp90 is not solely responsible for the maturation of the steroid hormone receptor but requires a specific set of binding associates it uses as co-chaperones [14, 18-21]. Collectively Hsp90 and its respective binding partners form dynamic multimeric assemblies in which the co-chaperones are replaced interchangeably at different stages of the steroid receptor cycle. In this cycle, the steroid receptor initially associates with Hsp40 and Hsp70, before being passed on to Hsp90 through intermediate binding interactions with the Heat shock Organizing Protein (HOP) [22]. Once the receptor is bound to the Hsp90 dimer, ATP-binding triggers the dissociation of HOP, leading to the subsequent binding of the Hsp90-receptor complex to other co-chaperones such as p23 and the 52 kDa immunophilin FK506-binding protein (FKBP52) [22]. Once in this mature complex, the receptor is primed for binding with its respective hormone ligand which in turn triggers a conformational change exposing the receptor's zinc-fingered DNA binding motifs to bind to the nuclear chromatin [23, 24].

As part of the mature chaperone complex, the small, acidic co-chaperone p23 binds directly to Hsp90 in a nucleotide-dependent fashion [25]. It is the smallest component in the Hsp90 machinery (M_r_ 18,000 - 25,000) and was first discovered in complex with Hsp90 and the progesterone receptor [26, 27]. The complete cellular function of p23 has not been fully elucidated. However, in the context of steroid hormone receptor regulation, the binding of p23 to Hsp90 is believed to stabilize the ATP-bound state of Hsp90 by obstructing the nucleotide-binding domains [28, 29]. A recent crystal structure of an Hsp90_2_-p23_2_ complex has confirmed this interaction in which p23 is seen bound to the amino-termini of Hsp90 through hydrophobic interactions [28]. As a result, p23 stabilizes the conformational changes induced by ATP binding which triggers the subsequent binding of immunophilin proteins and the activation of the steroid hormone receptor to its high-affinity ligand-binding state [30-33].

FKBP52 is a large peptidyl-prolyl *cis*-trans isomerase (PPIase) immunophilin protein that carries the ability to bind to immunosuppressive drugs. Structurally, FKBP52 consists of carboxy- and amino-termini that each have distinct roles in steroid hormone receptor regulation. A tetratricopeptide repeat domain (TPR) at the carboxy-terminus characterizes the binding surface for the well-defined -EEVD sequence motif at the carboxy-terminus of Hsp90 [34-37]. However, FKBP52 may also interact with the middle-domain (MD) of Hsp90 at a hydrophobic patch surrounding Trp300 [38-40] as seen with the Cdk4 kinase [41]. The amino-terminus of FKBP52 is highlighted by the FK-1 and FK-2 domains. Of particular interest, the FK-1 domain is believed to be largely responsible for FKBP52-mediated potentiation of receptor efficiency by providing the binding surface for the receptor [34, 42, 43]. Mutagenesis studies have indicated this to be true for point mutations in and around the FK-1 domain. These findings reveal that FKBP52-mediated potentiation of receptor activity is significantly abrogated in glucocorticoid (GR), androgen (AR) and progesterone (PR) receptor models [34, 42-45]. The physiological significance of FKBP52 has also been elucidated in knock out (KO) studies where male mice lacking the *Fkbp4* gene that encodes for FKBP52 display several developmental defects such as having ambiguous external genitalia and dysgenic prostate and seminal vesicles [44, 46].

In addition to p23 and FKBP52, HOP also plays a distinct role in steroid hormone receptor regulation by acting as an intermediary between Hsp70 and Hsp90. HOP recruits Hsp90 to interact with preexisting receptor-Hsp70 complexes, thus facilitating the well-articulated progression towards a fully mature receptor complex in which the receptor is primed for ligand-binding [7, 10, 11, 33, 47]. Like FKBP52, HOP is a TPR domain-containing protein and characteristically bind to Hsp90 in a very similar fashion [48]. FKBP52 and HOP are believed to both carry the ability to bind to Hsp90 at multiple sites, doing so in a competitive fashion while interacting at separate stages in the receptor cycle [11, 22, 32, 49, 50].

To date, the structural architecture of the intermolecular interactions between Hsp90 and its respected co-chaperone binding associates is still poorly understood, despite recent crystallographic data of the Hsp90_2_-p23_2_ complex [28]. In particular, our understanding of the interactions between Hsp90 with the various TPR domain-containing proteins remains unclear due to the lack of available structural data. In the work presented here, the stable formation of an Hsp90_2_-FKBP52_1_-p23_2_-HOP_2_ complex was detected by immunoprecipitation, time course dynamic light scattering (DLS) and electron microscopy (EM). Our results highlight the simultaneous binding of FKBP52 and HOP to the Hsp90 dimer independently of nucleotide and the p23 co-chaperone. Collectively these results provide evidence of a novel chaperone complex that likely plays a transient role in the regulation of fully mature steroid hormone receptors.

## RESULTS

### The purification of the p23, FKBP52, HOP and Hsp90 dimeric species

**Figure 1:**
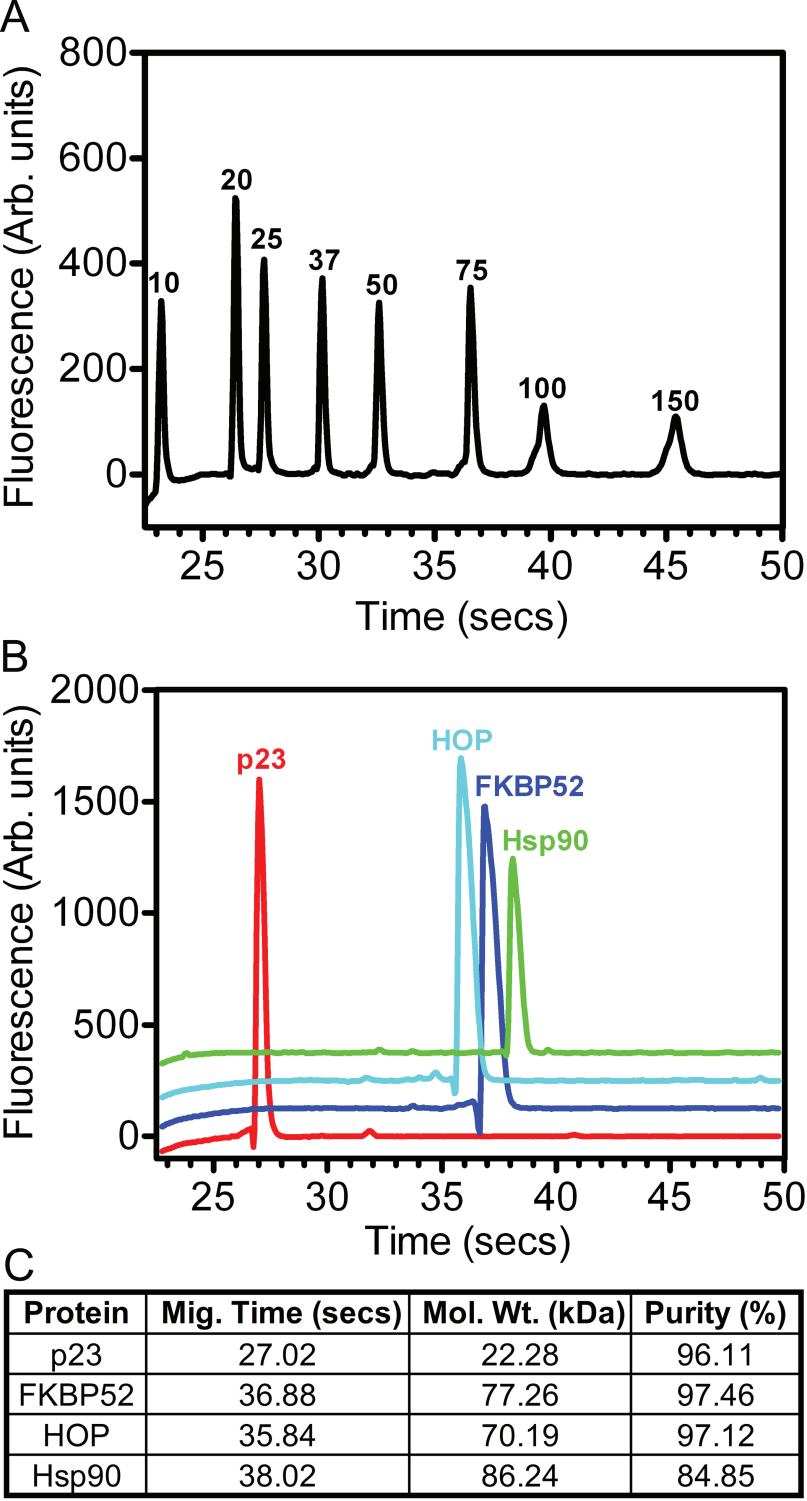
The purification of steroid hormone receptor chaperones and co-chaperones (A) A Laser excitation fluorescent detection chromatogram revealed the relative micro-fluidic migration positions of the molecular weight standards (values in kDa) depicted as a function of fluorescence versus time. (B) A similar laser excitation fluorescent detection chromatogram illustrating the migration positions of the p23 (red), FKBP52 (blue), HOP (cyan) and Hsp90 (green) proteins. The respective chromatographic spectra are staggered along the y-axis for purposes of clarity. (C) BIO-RAD Experion™ statistical analysis of the purified p23, FKBP52, HOP and Hsp90 proteins highlighting their respective migration times through the microfluidic channels of the Pro260 chips (seconds), as well as their estimated molecular weights (kDa) and relative degrees of homogeneity (%).

The constituent proteins for the formation of the Hsp90_2_-FKBP52_1_-p23_2_-HOP_2_ complex were individually purified using a number of column chromatographic techniques. Automated electrophoresis was used to analyze each target protein on the basis of molecular weight as a function of microfluidic channel migration (Figure 1A-B). The percentage of target protein relative to the total protein per sample was measured to highlight the relative homogeneity of each sample (Figure 1C).

**Figure 2:**
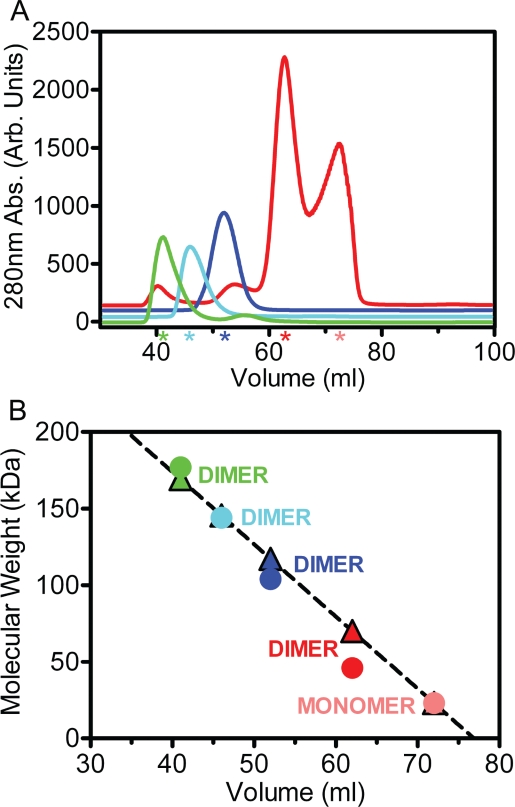
The existence of substrate dimers as observed by Sephacryl S200 size-exclusion chromatography (A) During analytical size-exclusion chromatography experiments, the p23 protein (red) eluted as two distinct peaks representative of dimer and monomer species. The FKBP52 (blue), HOP (cyan) and Hsp90 (green) proteins each eluted as single isolated peaks. Colored stars representative of each individual protein line the x-axis to highlight the elution points relative to volume. The two p23 peaks are individually represented by a red (dimer) and a light red (monomer) star. (B) A linear regression was done on the respective elution positions of protein standards with well-known molecular weights to interpolate the molecular weights of the target proteins from their elution positions. The colored triangles represent the interpolated molecular weight values derived from the linear regression (R2=0.88) while the colored circles denote molecular weight values derived from primary sequence information. Each protein is labeled to highlight its assumed multimeric conformation.

In the process of purifying the individual proteins prior to incorporating them into the Hsp90_2_-FKBP52_1_-p23_2_-HOP_2_ complex, it was revealed that each binding associate carries the ability to form a dimeric species. The elution positions of p23, FKBP52, HOP and Hsp90 in a size-exclusion (SEC) chromatogram relative to sizing controls were indicative of each respective protein being present as a dimer (Figure 2A). Additionally, p23 also eluted further downstream of its dimeric species as a monomer. The oligomeric states of each protein were validated against a linear regression derived from known standards and their well-resolved elution points from the Sephacryl S-200 column (Figure 2B).

### Stoichiometries of the Hsp90 interactions

**Figure 3:**
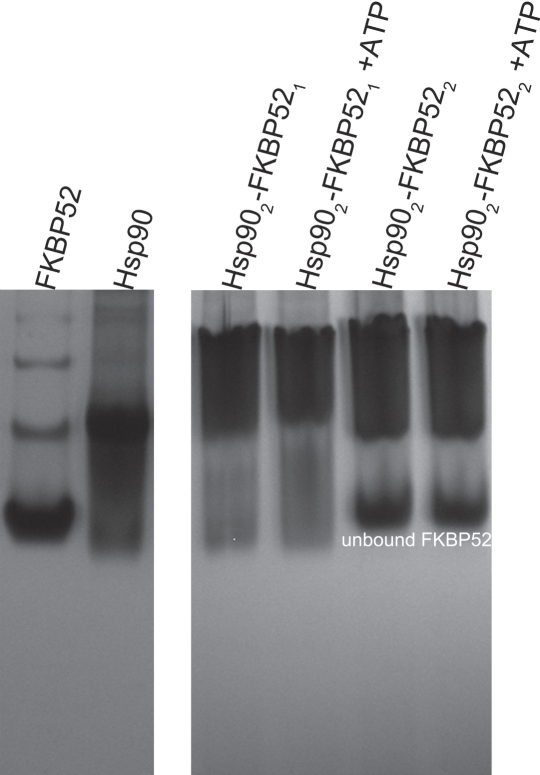
Stoichiometry of the Hsp90-FKBP52 interaction as determined by Native PAGE When FKBP52 was added to Hsp90 in a 1:2 ratio there was a noticeable upward shift in the position of the Hsp90 band in both the absence and presence of 5mM ATP, 20mM Na2MoO4. When added in a 2:2 ratio there fails to be an additional upward shift in the position of Hsp902-FKBP521 band, also illustrated by the large independent band of unbound FKBP52.

A series of Native PAGE experiments were conducted to assess the respective protein binding ratios of Hsp90 with p23 and the TPR domain-containing proteins. It was revealed that a significant amount of FKBP52 remained unbound when added to Hsp90 in a 2:2 molar ratio (Figure 3). For this reason, FKBP52 was added to Hsp90 in a 1:2 molar ratio in all subsequent experiments. The molar binding ratios HOP and p23 have with Hsp90 were indecipherable by Native-PAGE due to the indiscernible size difference between the HOP and Hsp90 dimers and the seemingly weak affinity Hsp90 has for p23. As an alternative, the HOP and p23 proteins were added to Hsp90 in a 2:2 or dimer-dimer molar ratio based on recently published isothermal titration calorimetry studies and crystallographic data [22, 28].

### FKBP52 and HOP can bind to Hsp90 simultaneously

**Figure 4:**
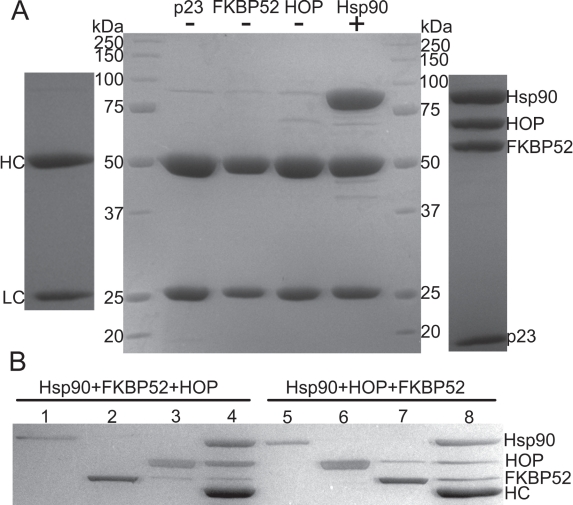
Co-immunoprecipitation experiments reveal the simultaneous binding interactions of Hsp90 with the two TPR domain-containing proteins FKBP52 and HOP (A) Hsp90-antibody specificity. Three negative controls (-) were performed in which the protein A Sepharose-Hsp90 antibody beads were incubated with 40μg the p23, FKBP52 and HOP proteins respectively. A positive control (+) incorporating 40μg Hsp90 into the antibody-bead mixture was also executed. Hsp90 was used as the bait protein for the subsequent experiments. Hsp90 antibody heavy (HC) and light (LC) chains (far left) as well as the anticipated distribution of the target proteins (far right). (B) The simultaneous binding of FKBP52 and HOP to Hsp90. Lanes 1-3 represent excess unbound protein in the initial washout fractions when Hsp90 (lane 1) was treated with a five-fold molar excess of FKBP52 (lane2) followed by a five-fold molar excess of HOP (lane 3) in a step-wise fashion. Lanes 5-7 represent a similar experiment in which HOP was added prior to FKBP52. Lanes 4 and 8 both represent noticeable amounts of Hsp902-FKBP521-HOP2 complex that were bound to the final antibody/Protein A-Sepharose bead pellets. Both the control experiments and the saturation experiments were resolved by 10% SDS-PAGE.

A series of immunoprecipitation experiments were performed to investigate the binding of p23, FKBP52 and HOP to the Hsp90 dimer using a monoclonal Hsp90 antibody in conjunction with Protein A-Sepharose beads. Initially, a series of control experiments were executed to highlight the direct specificity of the Hsp90 antibody (Figure 4A). Subsequently the simultaneous binding of FKBP52 and HOP to Hsp90 was tested revealing that the two proteins act independently of one another and in a non-competitive fashion (Figure 4B). Hsp90-bound antibody/Protein A beads were treated with a five-fold molar excess of FKBP52 protein to completely saturate the designated FKBP52 binding sites on the Hsp90 particles. After thorough washing, the beads containing FKBP52 bound to Hsp90 were treated with a five-fold molar excess of HOP to determine if HOP could still bind. Executing the co-IP experiment in a step-wise fashion ensured that the proteins bound to the final bead pellets were representative of FKBP52 and HOP binding at two different locations on Hsp90 and not a mixture of species in which the two proteins shared the same binding location (Figure 4B, lane 4). Sequential co-IP was also performed where the Hsp90-bound beads were first treated with a five-fold molar excess of HOP protein and then a five-fold molar excess of FKBP52 (Figure 4B, lane 8). It is important to note that in executing the co-IP experiments sequentially and in the presence of large molar excesses to saturate the respective binding sites on Hsp90 that the final bead pellet would reveal whether or not there is a competition for a single binding motif on Hsp90 when resolved by SDS-PAGE. Such a competition would be illustrated by the presence of only one band in addition to that of Hsp90, the identity of which would depend on which of the two TPR domain-containing proteins had the highest binding affinity for Hsp90. Interestingly the aforementioned competition was not observed here, illustrating that FKBP52 and HOP can bind to Hsp90 independently of one another.

A quantitative measure of binding stoichiometry of the Hsp90-HOP and Hsp90-FKBP52 interactions could not be derived from the intensity of the protein bands on the gel. However, the increased intensity of the final HOP protein bands compared to the final FKBP52 bands (Figure 4B, lanes 4 and 8) may suggest that the binding affinity that Hsp90 has for HOP is greater than that for FKBP52. It is also worth noting that the simultaneous binding of these TPR domain-containing proteins was independent of both ATP and p23 as both were absent in each experiment.

**Figure 5:**
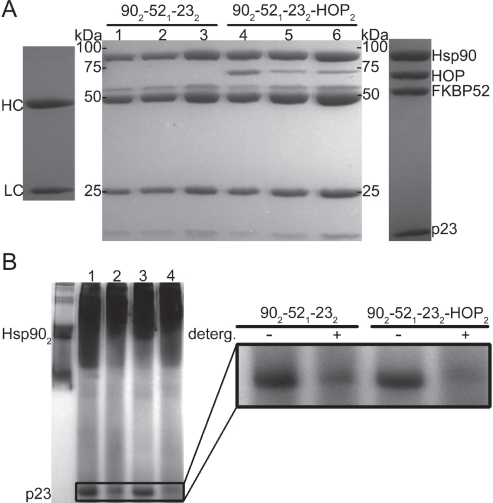
Co-immunoprecipitation and Native-PAGE experiments analyzing the relative degrees Hsp90_2_-FKBP52_1_-p23_2_ and Hsp90_2_-FKBP52_1_-p23_2_-HOP_2_ complex formation in the presence of varying buffering conditions (A) Lanes 1-3 represent the relative degree of Hsp90_2_-FKBP52_1_-p23_2_ formation in the absence of ATP, in the presence of ATP and with ATP plus 0.01% of a non-ionic detergent, respectively. Lanes 4-6 equate to the same buffering conditions withheld in lanes 1-3 but with the additional incorporation of HOP into the Hsp90_2_-FKBP52_1_-p23_2_-HOP_2_ complex. The SDS-PAGE image is surrounded by two additional lanes indicating the relative position of the Hsp90 antibody heavy (HC) and light (LC) chains (far left) as well as the anticipated distribution of the target proteins (far right). Results illustrated by 10% SDS-PAGE (B) Native-PAGE results revealing a greater degree of p23 incorporation into the respective Hsp902-FKBP521-p232 (Lanes 1-2) and Hsp902-FKBP521-p232-HOP2 (Lanes 3-4) complexes in the presence of 0.01% non-ionic dodecyl-maltopyranoside (Lanes 2 and 4).

In an effort to determine the effect ATP has on the FKBP52 and HOP interactions with Hsp90, additional experiments were performed in the presence of a 5mM ATP and 20mM Na_2_MoO_4_ (Figure 5A). ATP was added to trap the Hsp90 dimer in its ATP-bound conformation from which we could determine the relative effects conformational changes have on complex formation. As a result, the binding of FKBP52 to Hsp90 appeared to be unaffected by the addition of ATP in both the Hsp90_2_-FKBP52_1_-p23_2_ and Hsp90_2_-FKBP52_1_-p23_2_-HOP_2_ complexes (Figure 5A, lanes 1-2, 4-5). On the contrary, the relative degree of HOP binding to Hsp90 appears to be slightly diminished upon the addition of ATP (Figure 5A, lanes 4-5). Interestingly, complex formation and the binding of p23, FKBP52 and HOP to Hsp90 was slightly enhanced when the binding solution was augmented with a 0.01% dose of non-ionic detergent (Figure 5A, lanes 3,6) [51]. This phenomenon was also captured in Native-PAGE experiments in which the addition of non-ionic detergent appears to lead to a greater degree of Hsp90_2_-FKBP52_1_-P23_2_ and Hsp90_2_-FKBP52_1_-p23_2_-HOP_2_ complex formation as indicated by the relative disappearance of the independent p23 band (Figure 5B). This is likely explained by the non-ionic detergent making the hydrophobic interaction between Hsp90 and p23 more thermodynamically favorable.

**Figure 6:**
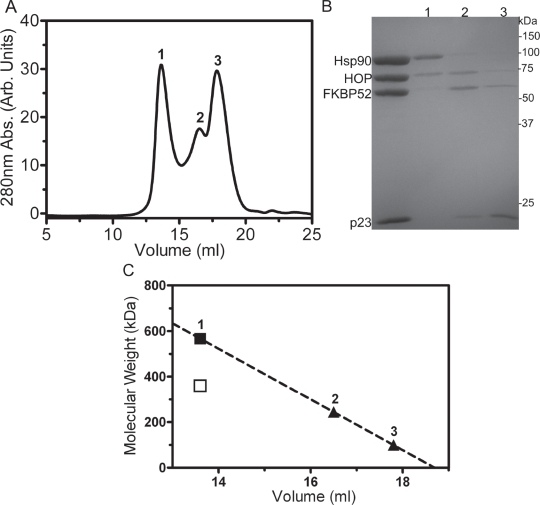
Analytical Superose6 size-exclusion chromatography (A) Size-exclusion chromatogram highlighting the separation of the Hsp90_2_-FKBP52_1_-HOP_2_ complex (peak 1) from unbound constituents (peaks 2 and 3). (B) 10% SDS-PAGE results of the three respective chromatographic peaks. (C) A linear regression of the Superose6 elution positions of protein standards was used to interpolate the molecular weights of the proteins eluting within the three peaks. The solid square and triangles represent the interpolated molecular weight values of the Hsp90_2_-FKBP52_1_-HOP_2_ complex and unbound constituents derived from the linear regression (R2=0.98). The hollow square denotes the molecular weight value of the Hsp90_2_-FKBP52_1_-HOP_2_ complex derived from primary sequence information

To further illustrate the simultaneous binding of FKBP52 and HOP to Hsp90, the Hsp90_2_-FKBP52_1_-p23_2_-HOP_2_ complex was passed through a Superose6 size-exclusion column (Figure 6A). Only a small fraction of Hsp90_2_-FKBP52_1_ -HOP_2_ complex was able to survive the relatively harsh separation process as a result of the dynamic nature of its formation (Figure 6B-lane 1). Two additional peaks eluted downstream of peak one likely corresponding to unbound constituents (Figure 6B-lanes 2 and 3). The molecular weight values of the protein species occupying the three chromatographic peaks was estimated to be approximately 575, 244 and 100 kDa respectively, when interpolated from a linear regression of molecular weight standards and their well-established elution positions from the Superose6 column (Figure 6C). p23 did not stay in complex with the other components due to the fragility of the Hsp90-p23 interaction in the absence of ATP. Analytical size-exclusion chromatography could not be performed in the presence of ATP without the final column flow becoming occluded. This may have resulted from the formation of large aggregates induced by conformational changes upon the addition of ATP or at the hand of the negatively charged nucleotide binding and accumulating onto the positively charged column matrix.

### Hsp902-FKBP521-p232-HOP2 complex formation

**Figure 7:**
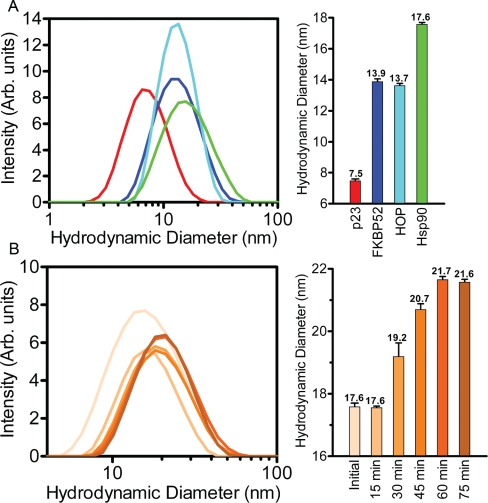
Hsp90_2_-FKBP52_1_-p232-HOP_2_ complex formation revealed by DLS (A) Dynamic light scattering spectra of the p23 (red), FKBP52 (blue), HOP (cyan) and Hsp90 (green) proteins illustrate diametric values that range between 7.6 and 17.6 nm as depicted on a logarithmic x-axis. Each individual constituent protein had a measured polydispersity values between 0.36-0.49.(B) The lateral shift in hydrodynamic diameter (increase in size) of the Hsp90_2_-FKBP52_1_-p23_2_-HOP_2_ complex (contrasting shades of orange) over the course of a 75 minute incubation at 30°C. Each individual sub-panel contains a bar graph representation of the DLS measurements outlined in the respective colors. Error bars depict the standard deviation within a 95% confidence interval.

Dynamic Light Scattering was used to track the formation of the Hsp90_2_-FKBP52_1_-p23_2_-HOP_2_ complex as a function of the progressive increase in the hydrodynamic diameter (d. nm) of the complex. Initially each of the binding associates was measured separately to provide a baseline of their individual oligomeric states (Figure 7A). p23 had the smallest hydrodynamic diameter reading of 7.5 nm while FKBP52 and HOP revealed very similar measurements of 13.9 and 13.7 nm respectively. Based on these results and the direct correlation between molecular weight and the volume of spatial occupancy, p23 was found to be present in its monomeric form while FKBP52 and HOP were found as dimers. The Hsp90 dimer was subjected to DLS analysis resulting in a measured diameter of 17.6nm. DLS measurements of Hsp90 were executed with and without ATP in an effort to capture differences in the hydrodynamic diameter of the ‘closed’ and ‘open’ conformations. However, the degree of variance between the two readings was too small to be regarded as outside of the range of instrumental accuracy.

To investigate the progressive formation of the Hsp90_2_-FKBP52_1_-p23_2_-HOP_2_ complex as a function of time, DLS measurements were taken over a 75 minute incubation period in the presence of 5mM ATP, 20mM Na_2_MoO_4_ and 0.01% non-ionic detergent. The shift from unoccupied Hsp90 through to the complete complex occurred in the first 60 minutes with the hydrodynamic diameter increasing from 17.6 to 21.7 nm (Figure 7B). More importantly this increase in diameter correlates with a large volumetric increase as the hydrodynamic diameter and volume are exponentially related (V=4/3 r3). Interestingly, the degree of complex formation was stabilized within the first 60 minutes of the incubation. The hydrodynamic diameter of the complex did not increase after 60 minutes.

**Figure 8:**
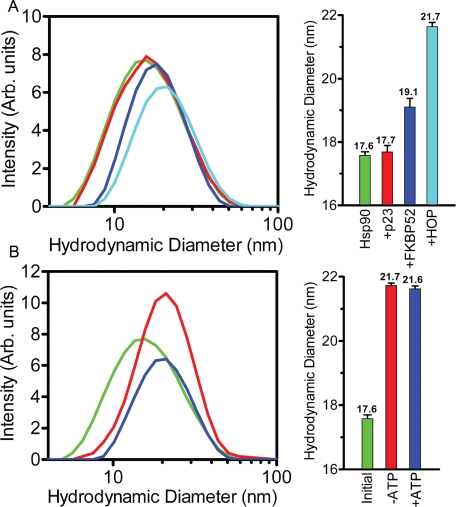
Step-wise and nucleotide-based formation of the Hsp90_2_-FKBP52_1_-p23_2_-HOP_2_ complex analyzed by DLS (A) DLS captured the incremental rightward shift in increasing hydrodynamic diameter as the individual components of the complex were added to Hsp90 (green) in a step-wise fashion. p23 was first added to Hsp90 to yield the Hsp90_2_-p23_2_ complex with a polydispersity value of 0.42 (+/− 0.11) (red). In subsequent steps, FKBP5_2_ and HOP were added to form the Hsp90_2_-FKBP52_1_-p23_2_ (blue) and Hsp90_2_-FKBP52_1_-p23_2_-HOP_2_ (cyan) complexes with polydispersity readings of 0.34 (+/− 0.12) and 0.40 (+/− 0.10) respectively. (B) The shift in hydrodynamic diameter of the Hsp90_2_-FKBP52_1_-p23_2_-HOP_2_ complex from the initial unbound Hsp90 (green) through to the complete complex after 60 minutes in both the absence and presence of 5mM ATP, 20mM Na_2_MoO_4_ (red and blue respectively). The results depicted in both sub-panels (A and B, left) are graphed against a logarithmic x-axis. Additionally each individual sub-panel contains a bar graph representation of the DLS measurements outlined in the respective colors. Error bars depict the standard deviation within a 95% confidence interval.

The formation of the Hsp90_2_-FKBP52_1_-p23_2_-HOP_2_ complex was also followed in a step-wise experiment revealing a gradual increase in the hydrodynamic diameter as more constituent proteins were added to Hsp90 (Figure 8A). The addition of p23 to Hsp90 in a 2:2 ratio resulted in a slight increase from 17.6 to 17.7 nm. This seemingly insignificant increase is likely the result of the tight packing of the p23 molecules to the amino-termini of the Hsp90 dimer as indicated by recent crystallographic data [28] and as supported by the Co-IP results presented here. The incorporation of FKBP52 into the Hsp90_2_-p23_2_ complex mixture resulted in an additional increase of 1.4 nm from 17.7 to 19.1 nm. More notable was the large increase in hydrodynamic diameter from the 19.1 nm Hsp90_2_-FKBP52_1_-p23_2_ complex to the 21.7 nm Hsp90_2_-FKBP52_1_-p23_2_-HOP_2_ complex (Figure 8A-right). The 2.6 nm increase in hydrodynamic diameter resulting from the addition of HOP to the Hsp90_2_-FKBP52_1_-p23_2_ complex indicated that HOP most likely bound to Hsp90 as a dimer. Interestingly like HOP, FKBP52 also exists as a dimer in solution but seemingly dissociates and binds to Hsp90 as a monomer whereas HOP remains bound as a dimer. Additionally, the degree of complex formation was analyzed as a function of nucleotide availability revealing unchanged diameter readings even when the sample was deprived of ATP (Figure 8B). This was further supported by similar polydispersity readings of 0.47 (+/− 0.185) and 0.45 (+/− 0.10) for the nucleotide-free and ATP-bound systems. As a control experiment, Hsp90 was analyzed in the presence of a 1:1 molar ratio of bovine serum albumin (BSA) of which it was no affinity for, to insure that the respective binding interactions with p23, FKBP52 and HOP were specific (Supplementary Figure 1).

In order to visualize the Hsp90_2_-FKBP52_1_-p23_2_-HOP_2_ complex, a sample of the complex was negatively-stained with 2% methyl-amine tungstate and examined by TEM (Supplementary Figure 2). At 100,000 × magnification the general shape of target particles within the micrographs can be characterized as elliptical and very reminiscent of the Hsp90_2_-p23_2_ X-ray structure [28]. As anticipated the particles representative of the Hsp90_2_-FKBP52_1_-p23_2_-HOP_2_ complex are noticeably larger than the unbound constituent proteins seen in the background. Some particles are oriented such that the separation in between the two Hsp90 monomers is visible and heavily stained (Supplementary Figure 2B). Other particles are oriented such that a noticeable amount of bulging density is seen protruding from one end of the Hsp90 dimer. We postulate that these protrusions are occupied by the binding of the FKBP52 and/or HOP TPR domain-containing proteins. It is worth noting that the binding of p23, FKBP52 and HOP appears to hold Hsp90 in its ‘closed’ conformation. This conformational change is clearly visible when particles of the complex are compared to that of unbound Hsp90 (Supplementary Figure 2C).

## DISCUSSION

In previous studies the human Hsp90 has been found to exhibit selective behavior for the binding of only one TPR domain-containing protein at a time [11, 49, 50]. This behavior has also been observed for the yeast immunophilin and HOP homologues cpr6 and Sti1 [30, 32]. In the work presented here, a number of biochemical and structural techniques were used to reveal the simultaneous binding of two TPR proteins to the Hsp90 dimer. The binding of FKBP52 and HOP to Hsp90 appears to be extremely stable and can be facilitated under a number of environmental conditions as indicated by co-IP, DLS, Native PAGE and electron microscopy experiments. Yeast studies have postulated that the binding of the p23 homologue Sba1 to Hsc82 is required prior to facilitating the binding of immunophilins [30, 32]. However, the co-IP, Native PAGE, EM and analytical size-exclusion chromatography results presented here indicate that the Hsp90-FKBP52 interaction occurs independently of p23 and can occur readily in the absence of ATP. Apart from FKBP52, HOP appears to interact with Hsp90 in a non-competitive fashion. Mutagenesis studies indicate that the binding of HOP to Hsp90 is nucleotide-independent, however this interaction can be weakened by the conformational changes induced by Hsp90's adaptation into the ATP-bound state [22]. The immunoprecipitation results presented here support this notion to a limited extent; however, DLS results indicate that the Hsp90-HOP interaction is also quite stable in the presence of 5mM ATP and 20mM Na_2_MoO_4_. HOP also interacts with Hsp90 in the presence and absence of p23. These results strikingly contradict previous findings that stipulate that the binding of p23 occludes or alters the stability of the binding site for HOP on Hsp90 [11].

The stoichiometry and location of the interactions between FKBP52 and HOP with Hsp90 has previously been unclear and has lead to a number of ambiguities. While all of the substrates maintain the ability to dimerize in solution, co-crystallization experiments support the notion that FKBP52 binds to the -EEVD peptide located at the far carboxy-terminus of each Hsp90 in a 1:2 molar ratio [35]. This hypothesis is further supported the Native PAGE results presented here in which a 2:2 molar mixture of FKBP52 to Hsp90 resulted in a large fraction of unbound immunophilin compared to when the two entities were added in a 1:2 ratio. Much like FKBP52, HOP also has binding affinity for the -EEVD motif [36] and can bind alternatively to the hydrophobic middle-domain of Hsp90 [32, 39, 40]. The Hsp90-HOP interaction likely occurs in a 2:2 ratio as indicated step-wise DLS results presented here and by other biochemical analyses [22, 30, 52]. The definitive stoichiometric binding ratios of the TPR domain-containing proteins with Hsp90 could not be resolved by our analytical size-exclusion chromatography experiments due to the strenuous nature of SEC separations. This issue can potentially be addressed through the use of ion mobility-mass spectrometric analysis (IM-MS), which has been used to reveal the subunit architecture of multimeric protein complexes of the bacterial replisome [53]. Additionally it is enticing to speculate over the Hsp90 binding positions of the FKBP52 and HOP proteins based on the particles in the presented EM micrographs. However, further structural investigations are required to reveal the arrangement of these intermolecular interactions.

**Figure 9:**
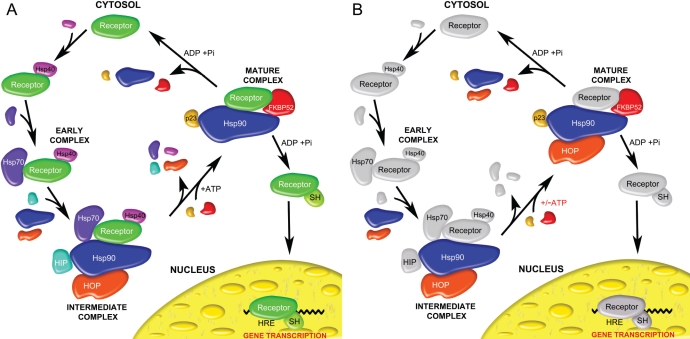
General models of the chaperone-assisted regulatory cycle of steroid hormone receptors (A) A cartoon schematic based on previous work hypothetically outlining the proteins involved in early, intermediate and mature complexes as the steroid hormone receptor is primed for binding with steroid hormone (SH) and an interaction with the hormone response elements (HRE) of the nucleus [22, 55, 59]. (B) Our newly proposed mechanism in which HOP offers stability to a receptor-Hsp90-FKBP52-p23 heterocomplex in the absence of the Hsp40, Hsp70 and HIP proteins. Colored proteins indicate those that were under investigation in this study.

In a recent model of the chaperone-mediated regulatory cycle of the steroid hormone receptor it has been postulated that there exists an intermediate assembly that is comprised of client protein (receptor) bound to the Hsp90, Hsp70, Hsp40, HIP and HOP proteins [54] (Figure 9A). Upon the binding of ATP to Hsp90, the Hsp70, Hsp40, HIP and HOP proteins dissociate from Hsp90 and allow for the binding of immunophilins like FKBP52 or cyclophilin 40, and the p23 co-chaperone. This Hsp90_2_-FKBP52_1_-p23_2_ assembly characterizes a mature complex that is competent to facilitate hormone binding [22, 55]. While this model seems quite promising, the formation of the Hsp90_2_-FKBP52_1_-p23_2_-HOP_2_ complex revealed here indicates that the intermediary role played by HOP is likely more substantial than previously described. HOP can remain bound to Hsp90 in the presence of immunophilin and p23 and likely offers stabilizing effects that were previously offered to the client protein by the proteins of the intermediate complex (Figure 9B). Once stabilized by Hsp90, HOP, FKBP52 and p23, it seems likely that ATP hydrolysis within Hsp90 triggers the dissociation of ligand-bound receptor for direct transport to the nucleus. However, it remains unclear as to whether or not the ligand-bound steroid receptor travels alone to the nucleus or with the accompaniment of Hsp90. Furthermore its remains to be seen whether a steroid receptor/Hsp90 heterocomplex can permeate through the nuclear pores as has been demonstrated in the mineralocorticoid receptor system [56].

Despite the intrigue of the Hsp90_2_-FKBP52_1_-p23_2_-HOP_2_ complex and its possible involvement in the regulation of steroid hormone signal transduction, the importance of its formation remains to be seen in the presence of purified hormone receptor. Addressing this issue is a daunting task largely due to the difficulties associated with producing functional amounts of stable client protein and the transient nature of the receptor-chaperone binding interactions. It is likely that FKBP52 is present in the mature receptor-chaperone complex as the FK-1 domain is believed to be largely responsible for FKBP52-mediated potentiation of receptor efficiency by providing the binding surface for the receptor [34, 42, 43]. However, whether p23 and/or HOP remain bound to Hsp90 in the presence of the receptor remains unclear. Subsequent experiments using a purified steroid receptor sample are required to shed light on this question in hopes of further elucidating the roles p23, FKBP52 and HOP play in Hsp90-mediated steroid hormone receptor regulation.

## METHODS

### Protein expression and purification

The human FKBP52, HOP and Hsp90α proteins were expressed as amino-terminal 6x-Histidine-tagged constructs from the vectors pET28b-FKBP52, pET28b-HOP, and pET28b-Hsp90α, respectively. The human p23 co-chaperone was expressed as an untagged construct from the pET23-p23 vector. *Escherichia coli* BL21 (DE3) transformants were grown at 37°C to an *A*600nm optical density of 0.8 before protein expression was induced with 1mM IPTG at 30°C for several hours. The cells were harvested by centrifugation and lysed in a 50mM Hepes, 10mM EDTA lysis, 1mM PMSF buffer pH 7.5 in conjunction with treatment with hen egg white lysozyme (Sigma) and multiple freeze/thaw cycles.

The 6x-His-tagged FKBP52, HOP and Hsp90 proteins were initially purified using a Ni2+ Sepharose HisTrap FF column (GE). The column was equilibrated and washed with 50mM Hepes, 100mM NaCl, 10mM MgCl_2_, 20mM Imidazole, 1mM PMSF, 0.02% NaN_3_ (pH 7.5) and the respective proteins were eluted with a 250mM Imidazole step gradient. The target fractions for each of the respective proteins were further purified on a HiLoad Q-Sepharose 26/10 anion-exchange column (Amersham) that was equilibrated with 50mM Hepes, 10mM MgCl_2_, 1mM DTT, 1mM PMSF, 0.02% NaN_3_ (pH 7.5). The proteins were each eluted over a 400ml 0-600mM NaCl linear gradient [57]. Additionally, the HOP and Hsp90 proteins were passed through a HiPrep Sephacryl S-200 size-exclusion column (Amersham) that was equilibrated with 50mM Hepes, 150mM NaCl, 50mM KCl, 10mM MgCl_2_, 1mM DTT, 1mM PMSF, 0.02% NaN_3_ (pH 7.5). Despite having near absolute sample homogeneity after passage over the Q-Sepharose anion-exchange column, the FKBP52 sample was also subjected to treatment through the Sephacryl S-200 column for analysis of its multimeric conformational state.

The human p23 protein was initially purified over a quad-stack of 5ml DEAE FF anion-exchange columns (Amersham). The columns were equilibrated with 50mM Hepes, 10mM MgCl_2_, 1mM DTT, 1mM PMSF, 0.02% NaN_3_ (pH 7.5) and the target protein was eluted over a 100ml 0-600mM NaCl linear gradient. The target fractions were then passed through a HiLoad Q-Sepharose column as previously described. p23 was then further purified on a HiPrep Sephacryl S-200 size-exclusion column as previously described, eluting in two distinct peaks corresponding to the dimeric and monomeric species. The chromatography standards used in the linear regression included IgG (M_r_ 160 kDa), BSA (M_r_ 67), β-lactoglobulin (M_r_ 35), lysozyme (M_r_ 15 kDa), cytochrome C (M_r_ 12.4 kDa) and cytidine (M_r_ 240 Da) which elute at 48, 56, 66, 74, 78 and 120 ml, respectively.

All proteins were dialyzed extensively against 50mM Hepes 50mM KCl 10mM MgCl_2_ (pH 7.4) prior to storage at −80°C. The homogeneity of the proteins was determined by SDS-PAGE and the Experion™ Automated Electrophoresis station (BIO-RAD). Protein concentrations were determined spectrophotometrically via BCA assay.

### Native-PAGE analysis

All individual protein samples and protein mixtures were resolved by 7.5% polyacrylamide in which no detergents, reducing or denaturing agents were used in any of the resolving, stacking or loading buffers. 5μg of total protein was loaded into each well with the Hsp90, FKBP52, HOP and p23 proteins being mixed together in their designated 2:1:2:2 molar ratios in the respective experiments. All protein complex mixtures were incubated at 30°C for 60 minutes as indicated in previous studies [7, 51, 55]. The samples were not treated with heat prior to loading and all electrophoresis was done on ice

### Immunoprecipitation pull-down assays

In each experiment 25μl of Protein A-Sepharose beads (GE) was equilibrated with 40μg of monoclonal Hsp90 (H90-10) antibody at room temperature for 30 minutes in a 50mM Hepes, 50mM KCl, 10mM MgCl_2_, 1mM DTT solution (pH 7.4). The beads were washed multiple times and recovered by centrifugation prior to loading 40μg of Hsp90α bait protein. To test the specificity of the Hsp90 antibody 40μg of p23, FKBP52 and HOP were alternatively added in separate negative control reactions. The antibody/protein mixtures were incubated for 1 hour on ice with the pellets being harvested by centrifugation and washed multiple times in the previously described buffering system. To assess the simultaneous binding of FKBP52 and HOP to Hsp90, Hsp90-bound antibody/Protein A beads were saturated with five-fold molar excesses of each of the respective proteins in a step-wise fashion. At each step the protein mixtures were incubated at 30°C for 60 minutes with gentle shaking occurring every five minutes. After the first incubation the beads were washed extensively prior to another five-fold molar excess addition of the second TPR domain-containing protein. In additional experiments the p23, FKBP52, and HOP proteins were added to the Hsp90 bait in equimolar amounts according to the 2:1:2:2 molar binding ratios in the Hsp90-FKBP52-p23-HOP complex. The protein mixtures were incubated at 30°C for 60 minutes with gentle shaking occurring every 5 minutes. The buffering system was augmented with a 5mM ATP, 20mM Na_2_MoO_4_ supplement and a 0.01% dose of the non-ionic detergent dodecyl-maltopyranoside in respective experiments. All unbound proteins were washed away from the Hsp90 bait with multiple washes and the harvested beads carrying Hsp90 and the bound complex constituents were resolved by 10% SDS-PAGE.

### Analytical Size-Exclusion Chromatography

A 200 μl sample of 18μg/μl Hsp90_2_-FKBP52_1_-p23_2_-HOP_2_ complex was passed through a Superose6 size-exclusion column (Amersham). The complex was separated from unbound constituent proteins over a 50ml elution period using a 50mM Hepes, 50mM KCl, 10mM MgCl_2_, 1mM PMSF, 1Mm DTT, 0.02% NaN_3_ buffer. The chromatography results were visualized by 10% SDS-PAGE. The chromatography standards used in the linear regression included thyroglobulin (M_r_ 669 kDa), ferritin (M_r_ 440), aldolase (M_r_ 158), ovalbumin (M_r_ 43 kDa), carbonic anhydrase (M_r_ 29 kDa) and RNAse A (M_r_ 13.7 kDa) which elute at 13, 14.5, 17, 18, 18.5 and 19 ml, respectively.

### Dynamic Light Scattering

The diameter of the solvation layers surrounding each of the constituent proteins (hydrodynamic diameter) was measured in the Zetasizer Nano series system (Malvern). 0.02mg/ml samples of each of the individual proteins were measured at 30°C with measurements occurring every 15 seconds for a total of 50 measurements with the total incubation time lasting 60 minutes. Complex formation as a function of time was analyzed over the course of 90 minutes in a 50mM Hepes, 50mM KCl, 10mM MgCl_2_, 1Mm DTT solution (pH 7.4) with and without the addition of the 5mM ATP, 20mM Na_2_MoO_4_ and detergent supplements. The step-wise formation of the Hsp90_2_-FKBP52_1_-p23_2_-HOP_2_ complex was analyzed in the aforementioned buffer with the ATP, Na_2_MoO_4_ and detergent supplements in which measurements were taken over the course of 60 minutes at each step. The degree of non-specific binding with Hsp90 was assessed with a 0.02mg/ml sample of Hsp90 in a 1:1 molar ratio with bovine serum albumin in which measurements were taken over a 60 minute time course. The accuracy and reproducibility of each experiment was analyzed by standard deviation within a 95% confidence interval.

### Electron Microscopy

The p23, FKBP52, HOP and Hsp90 proteins were diluted down from their respective 7, 9, 8, and 9 mg/ml concentrations to a final complex concentration of 0.02 mg/ml by mixing the constituents together according to their molar ratios in the Hsp90_2_-FKBP52_1_-p23_2_-HOP_2_ complex. The individual proteins were incubated together at 30°C for 60 minutes as previously indicated in DLS experiments. The sample was blotted onto carbon grids and stained with 2% Methyl-amine tungstate. The images were collected on a Hitachi transmission electron microscope operating at a voltage of 80,000 V. CCD images were collected at 100,000x magnification. Individual particles were boxed from the micrograph using the BOXER program within the EMAN software suite [58]. As a reference images of individual unbound Hsp90 particles were prepared as previously described and were collected on a JEOL JEM 3200FS transmission electron microscope operating at 300,000 V. CCD images were collected at 80,000x magnification.

## SUPPLEMENTAL FIGURES




