# Supplementary figures and images for: Hsp90 can Accommodate the Simultaneous Binding of the FKBP52 and HOP Proteins
Source: Oncotarget. 2011 Feb 28;2(1-2):43–58. doi: 10.18632/oncotarget.225 (PMC3248148; doi:10.18632/oncotarget.225)

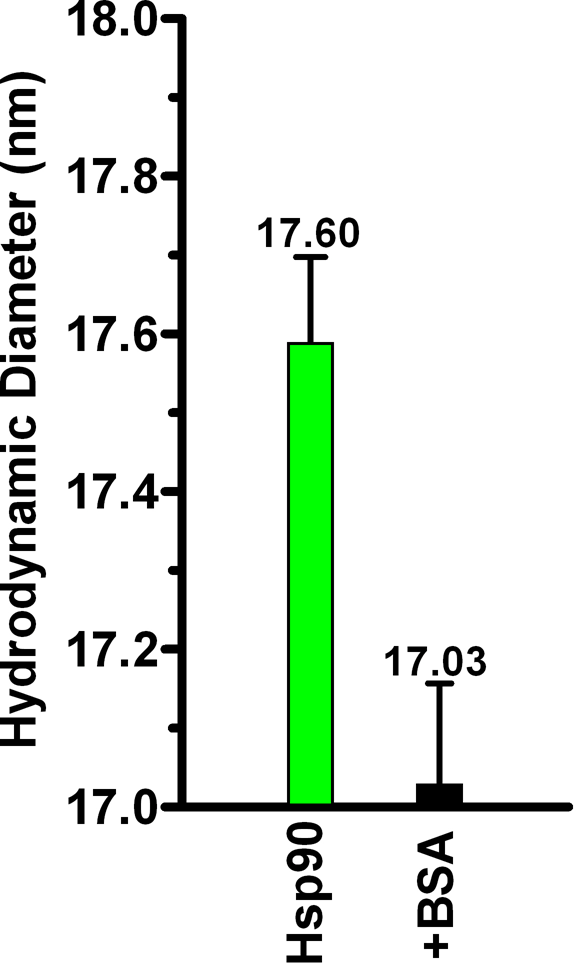

Supplement: Supplementary file 1 [file oncotarget-02-043-s001.tif]

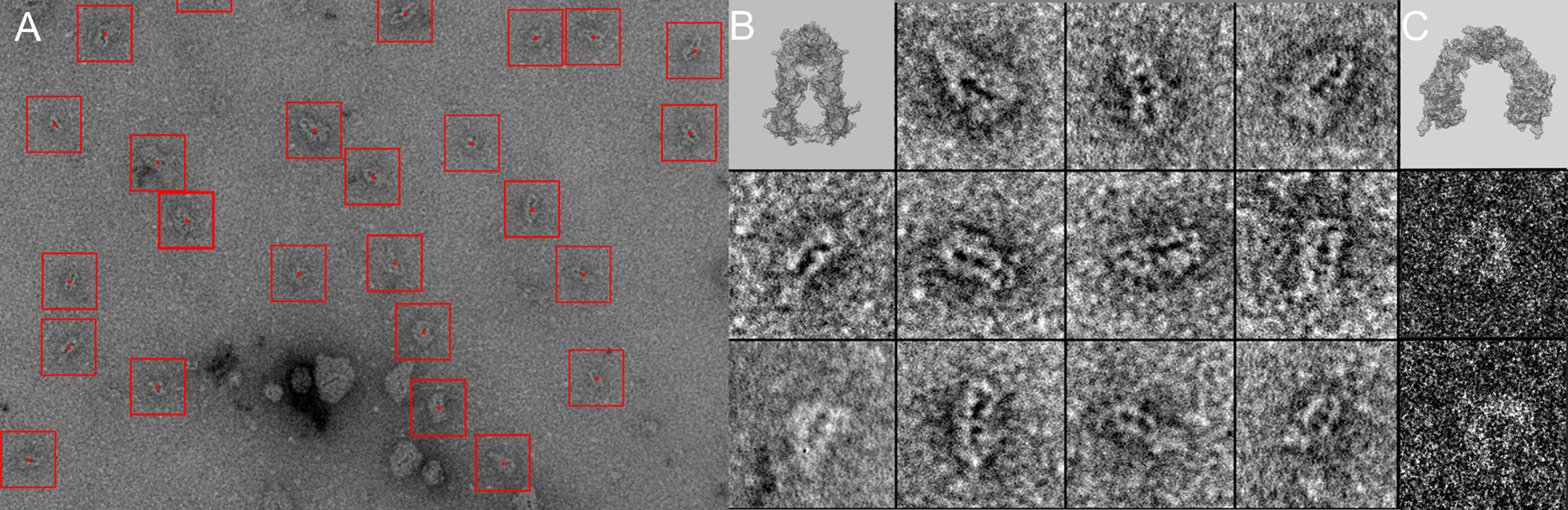

Supplement: Supplementary file 2 [file oncotarget-02-043-s002.tif]
